# Analysis of Yield of Eleutherosides B and E in *Acanthopanax divaricatus* and *A. koreanum* Grown with Varying Cultivation Methods

**DOI:** 10.1155/2014/515291

**Published:** 2014-07-21

**Authors:** Jeong Min Lee, Myoung-Hee Lee, Suk-Bok Pae, Ki-Won Oh, Chan-Sik Jung, In-Youl Baek, Sanghyun Lee

**Affiliations:** ^1^Department of Integrative Plant Science, Chung-Ang University, Anseong 456-756, Republic of Korea; ^2^Department of Functional Crops, National Institute of Crop Science, Rural Development Administration, Miryang 627-803, Republic of Korea

## Abstract

An analysis of the yield of eleutherosides B and E in *Acanthopanax divaricatus* and *A. koreanum* was performed using high performance liquid chromatography to evaluate production by different cultivation methods. In *A. divaricatus* and *A. koreanum*, the total content of eleutherosides B and E was 2.466–7.360 mg/g varying by plant section, 3.886–11.506 mg/g by pinching site, 3.655–10.083 mg/g by planting time, and 3.652–10.108 mg/g by fertilizer ratio. Thus the total content of eleutherosides B and E in *A. divaricatus* and *A. koreanum* differed depending on cultivation methods. These results present useful information for high eleutheroside content applications in *A. divaricatus* and *A. koreanum*. This information can affect selection of plant section and cultivation methods for nutraceutical, pharmaceutical, and cosmeceutical material development.

## 1. Introduction

*Acanthopanax* species are commonly known as Siberian ginseng, touch-me-not, devil's shrub, prickly, and wild pepper [1]. A diverse group of chemical compounds isolated from* Acanthopanax* species was named “eleutherosides.” The group of eleutherosides consists of eleutherosides A (daucosterol), B (syringin), B_1_ (isofraxidin-7-*O*-glucoside), B_2_, B_3_, B_4_ (sesamin), C (methyl-*α*-D-galactoside), D, E (syringaresinol-di-*O*-*β*-D-glucoside), E_1_ (syringaresinol-*O*-*β*-D-glucoside), E_2_ (episyringaresinol 4′′-*O*-*β*-D-glucoside), F, G, I, K, L, and M (hederasaponin B) [2–5]. Among the eleutherosides, eleutheroside E from* A. senticosus* has the most noticeable stimulant and antistress effects [6].

There have been many studies on the activities of eleutherosides B and E. Eleutherosides B and E have been reported to have protective effects against amyloid *β*(25–35)-induced neuritic atrophy in cultured rat cortical neurons [7], neuroprotective effects against transient global cerebral ischemia in rats [8], protective effects in dopaminergic neurons in Parkinson's disease mice [9], and antioxidant properties [10]. There have been many studies determining the effects of eleutherosides B and E. The quantitative analysis of eleutherosides B and E from* Acanthopanax* species has been reported from various parts of members of* Acanthopanax* species [11, 12], including* A. sessiliflorus* fruits and fermented wine made from them [13] and in the roots of* A. senticosus* [14].

*Acanthopanax* species are cultivated and grow wild in various areas in Korea, and the variety of their pharmacological effects has attracted consumers' interests. However, there are many difficulties in producing high quality* Acanthopanax* species, depending upon the location and method of culture, which affect different pharmacological ingredients [15].

This study, therefore, analyzed eleutherosides B and E in* A. divaricatus* and* A. koreanum*, depending on plant section and cultivation method, using high performance liquid chromatography (HPLC) with the goal of optimizing the content of eleutherosides B and E by cultivation method, to suggest efficient cultivation methods for* A. divaricatus* and* A. koreanum*.

## 2. Materials and Methods

### 2.1. Plant Materials

**Figure 1:**
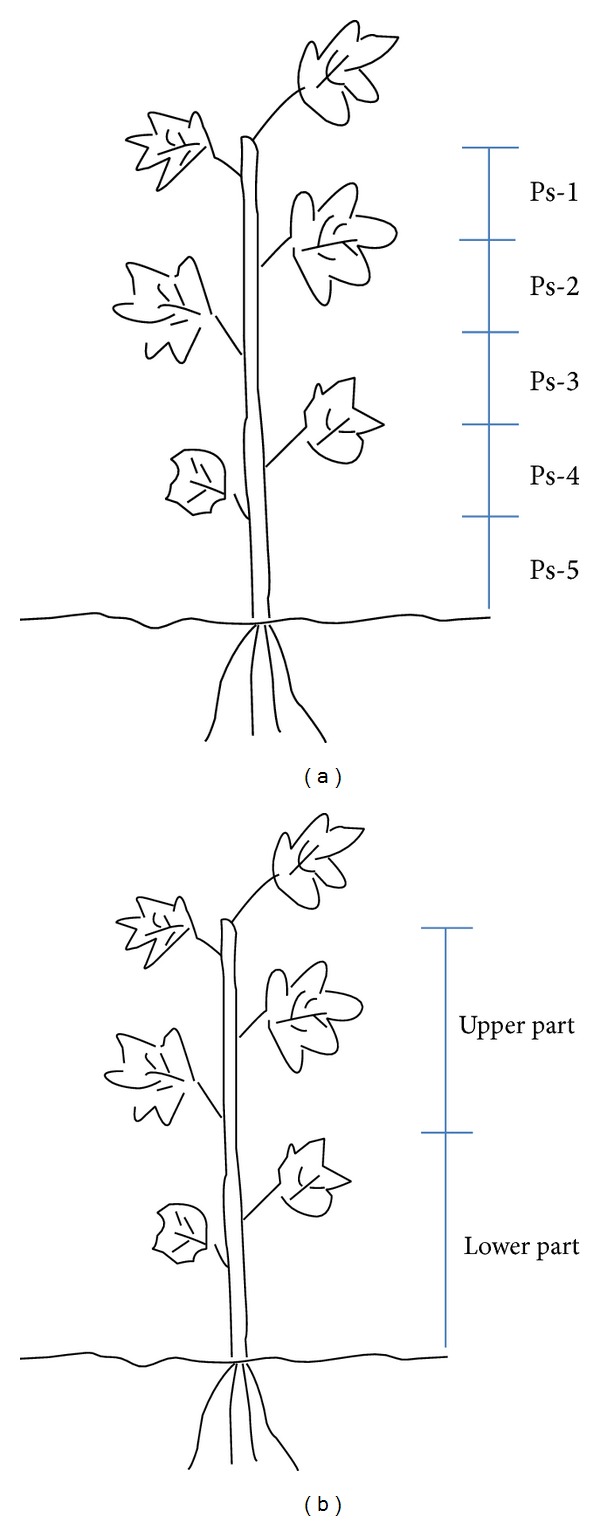
Sample collection fractions by plant section (a) and by upper and lower parts of plants (b).

*A. divaricatus* (Voucher number LEE11-1) and* A. koreanum* (Voucher number LEE11-2) were collected at Yeongcheon Agricultural Technology & Extension Center, Yeongcheon, Korea. Plant section (Ps) was designated as Ps-1, -2, -3, -4, or -5 (Figure 1). Different cultivation method criteria consisted of pinching site (30 and 60 cm), planting time (March 30, April 15, and April 30), and fertilizer ratio (N-P-K, 10.5-8.5-8.5: 50 kg/10a; 2N-P-K, 21-8.5-8.5: 50 kg/10a; N-2P-K, 10.5-17-8.5: 50 kg/10a; N-P-2K, 10.5-8.5-17: 50 kg/10a; 2N-2P-2K, 21-17-17: 50 kg/10a).

### 2.2. Cultivation Methods

Tilling was carried out on March 20, during which fully fermented compost (1,500 kg/10a) was added to the soil, and* A. divaricatus* and* A. koreanum* seedlings were planted on March 30. The compound fertilizer (2N-2P-2K, 21-17-17) fertilized the soil once in the June 10. The harvest time of all samples was February 13 of the following year. The cultivation conditions (2007) were 13.1°C average temperature and 1,142 mm average precipitation. The soil conditions were mature soil, pH 7.0, 3.4% soil organic content, 435 ppm available phosphate, and 0.36, 3.0, and 1.2 cmol^+^/kg of K, Ca, and Mg, respectively [16]. All samples were cultivated under the same above conditions.

### 2.3. Apparatus and Chemicals

Evaporation was conducted using an Eyela rotary evaporator system (Tokyo, Japan) under reflux* in vacuo*. HPLC chromatograms were recorded with an Agilent 1100 series HPLC (Waldbronn, Germany) equipped with Agilent 1100 series G1311A Quat pump and Agilent 1100 series G1315B detector. A Discovery C18 (4.6 × 250 mm, 5 *μ*m) column was purchased from Sigma-Aldrich Co. (PA, USA). Water and acetonitrile used in this research were of HPLC grade, and all other reagents were of analytical grade.

### 2.4. Preparation of Eleutherosides B (**1**) and E (**2**)

The air-dried powdered stems of* A. senticosus* were extracted with H_2_O. The extract was suspended in H_2_O and then partitioned successively with equal volumes of chloroform, ethyl acetate, and* n*-butanol. For qualitative and quantitative analysis, a combined eleutherosides B (CHCl_3_ : MeOH = 95 : 5) and E (CHCl_3_ : MeOH = 90 : 10) isolate was obtained by repeated column chromatography from the *n*-butanol fraction of stem of* A. senticosus* [10, 12].

Eleutheroside B (**1**): FAB-MS:* m/z* 373 [M+H]^+^; ^1^H-NMR (500 MHz, DMSO-*d*_6_): *δ* 6.73 (2H, s, H-2,6), 6.46 (1H, d,* J *= 15.9 Hz, H-7), 6.33 (1H, dt,* J* = 15.9, 5.1 Hz, H-8), 4.84 (1H, d,* J* = 7.5 Hz, glucosyl H-1), 4.11 (1H, dd,* J* = 5.1, 1.4 Hz, H-9a), 4.09 (1H, dd,* J* = 5.1, 1.4 Hz, H-9b), 3.77 (6H, s, 2 × OMe); ^13^C-NMR (125 MHz, DMSO-*d*_6_): *δ* 152.7 (C-3,5), 133.0 (C-4), 131.0 (C-7), 129.0 (C-8), 128.1 (C-1), 104.5 (C-2,6), 103.1 (Glc C-1), 77.4 (Glc C-5), 76.5 (Glc C-3), 74.9 (Glc C-2), 71.0 (Glc C-4), 62.0 (C-9), 60.5 (Glc C-6), 56.3 (OMe).

Eleutheroside E (**2**): FAB-MS:* m/z* 743 [M+H]^+^; ^1^H-NMR (500 MHz, DMSO-*d*_6_): *δ* 6.67 (4H, s, H-2′,6′), 4.88 (2H, d,* J* = 7.3 Hz, glucosyl H-1), 4.67 (2H, d,* J* = 3.6 Hz, H-2), 4.28 (2H, dd,* J* = 8.5, 6.6 Hz, H-4_eq_), 4.20 (2H, dd,* J* = 8.5, 3.0 Hz, H-4_ax_), 3.76 (12H, s, 4 × OMe), 3.19 (2H, m, H-1); ^13^C-NMR (125 MHz, DMSO-*d*_6_): *δ* 153.2 (C-3′,5′), 138.1 (C-4′), 134.1 (C-1′), 104.6 (C-2′,6′), 103.3 (Glc C-1), 85.7 (C-2), 77.5 (Glc C-5), 76.7 (Glc C-3), 74.5 (Glc C-2), 72.1 (C-4), 70.2 (Glc C-4), 61.2 (Glc C-6), 57.0 (OMe), 54.2 (C-1).

### 2.5. Sample Preparation

To analyze the eleutherosides B (**1**) and E (**2**) content in* A. divaricatus* and* A. koreanum*, 5 g of each of* A. divaricatus* and* A. koreanum* was extracted with 50% MeOH (3 × 100 mL) by reflux and evaporated* in vacuo*. The residue was dissolved in 1 mL of MeOH and filtered with a 0.45 *μ*m filter. The resulting solution was used for HPLC analysis.

### 2.6. HPLC Conditions

HPLC separation of eleutherosides B (**1**) and E (**2**) for qualitative and quantitative analysis was performed using a reverse phase system. A Discovery C18 (4.6 × 250 mm, 5 *μ*m) column was used with a mobile phase that consisted of water and acetonitrile. A gradient solvent system of water and acetonitrile (90 : 10 to 70 : 30 for 20 min) was used for the elution program. UV detection was conducted at 350 nm. The injection volume was 10 *μ*L and the flow rate was 1 mL/min. The temperature was maintained at 25°C. All injections were performed in triplicate.

### 2.7. Calibration Curve

A stock solution (1 mg/mL) of each eleutherosides B (**1**) and E (**2**) isolate was prepared in MeOH, and then the solution content was successively reduced to 50% to create different concentrations. The analyte contents were determined from the corresponding calibration curves. The calibration functions of the eleutherosides B (**1**) and E (**2**) isolate were calculated using the peak area (*Y*), concentration (*X*, *μ*g/10 *μ*L), and mean values (*n* = 3) ± standard deviation (SD).

### 2.8. Statistical Analysis

Data for each sample was expressed as mean ± SD. ANOVA using the SAS Enterprise Guide software was calculated and the significance between the means of each group was determined using Duncan's multiple test.

## 3. Results and Discussion

**Figure 2:**
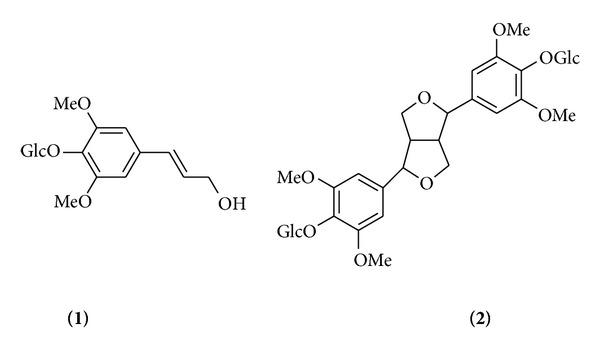
Chemical structures of eleutherosides B (**1**) and E (**2**).

Content analysis of eleutherosides B (**1**) and E (**2**) in* A. divaricatus* and* A. koreanum* by plant section and cultivation method was conducted by HPLC. Eleutherosides B (**1**) and E (**2**) (Figure 2) have previously been isolated from* A. senticosus*,* A. divaricatus*, and* A. koreanum* [12, 17, 18].

**Figure 3:**
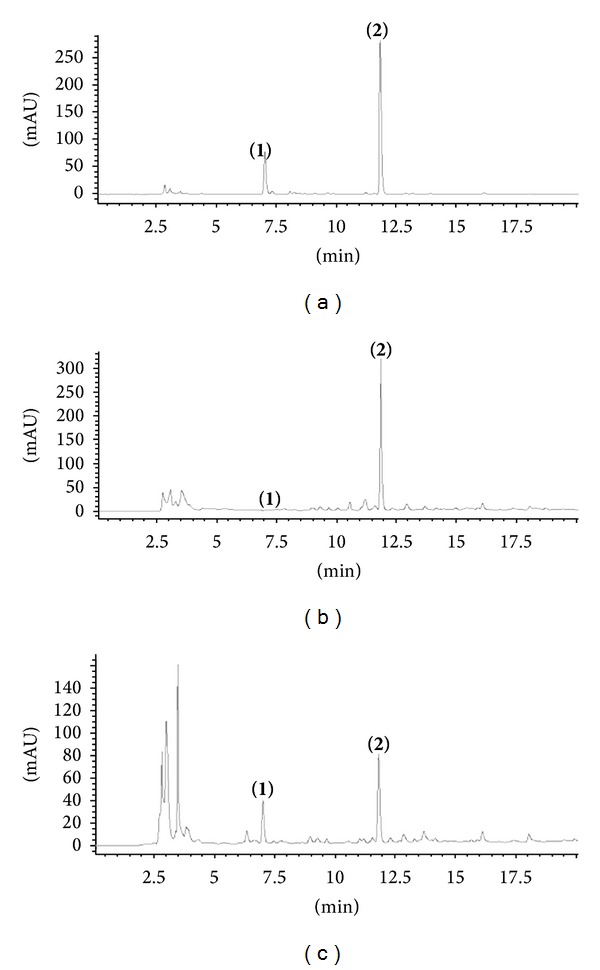
HPLC chromatograms of standards of eleutherosides B (**1**) and E (**2**) (a) and of the MeOH extracts from* A. divaricatus* grown with fertilizer ratio N-2P-K (b) and from* A. koreanum* grown with fertilizer ratio 2N-2P-2K (c).

HPLC was used to separate eleutherosides B (**1**) and E (**2**) for qualitative and quantitative analysis using a reverse phase system. HPLC conditions were selected to analyze eleutherosides B (**1**) and E (**2**) with good linearity (eleutheroside B, *r* ^2^ = 0.9999; eleutheroside E, *r* ^2^ = 0.9997). The contents of eleutherosides B (**1**) and E (**2**) in* A. divaricatus* and* A. koreanum* were determined, examining variations in plant section, pinching site, planting time, and fertilizer ratio, as described in Section 2. The HPLC chromatograms of eleutherosides B (**1**) and E (**2**) standards and of* A. divaricatus* and* A. koreanum* 50% MeOH extracts are shown in Figure 3.

The total eleutherosides B (**1**) and E (**2**) content in different plant sections (Ps-1 to -5) ranged from 2.466 to 5.841 mg/g in* A. divaricatus* and from 4.339 to 7.360 mg/g in* A. koreanum* (Table 1). The total eleutherosides B (**1**) and E (**2**) content in the upper part (Ps-1) was greater than that in the lower part (Ps-5) of* A. divaricatus*, butthe result in* A. koreanum* was the opposite. The total eleutherosides B (**1**) and E (**2**) content was the highest in Ps-1 of* A. divaricatus* and Ps-5 of* A. koreanum*.

The total eleutherosides B (**1**) and E (**2**) content was found to be similar in the upper and lower parts of* A. divaricatus* and* A. koreanum* when different cultivation methods were employed. Pinching (30 and 60 cm) yielded total eleutherosides B (**1**) and E (**2**) contents of 6.301–6.827 and 7.858–11.506 mg/g in the upper and lower parts of* A. divaricatus*, respectively, while in* A. koreanum*, the total eleutherosides B (**1**) and E (**2**) contents were 4.439–7.847 and 3.886–4.161 mg/g in the upper and lower parts, respectively (Table 2). When planting time was varied (March 30, April 15, and April 30), the total eleutherosides B (**1**) and E (**2**) contents were found to be 4.290–6.526 and 3.769–10.083 mg/g in the upper and lower parts of* A. divaricatus*, respectively, while the total eleutherosides B (**1**) and E (**2**) contents were found to be 4.546–5.693 and 3.655–6.529 mg/g in the upper and lower parts of* A. koreanum*, respectively (Table 3). When fertilizer ratio was varied (N-P-K, 2N-P-K, N-2P-K, N-P-2K, and 2N-2P-2K), the total eleutherosides B (**1**) and E (**2**) contents were found to range from 4.417 to 6.905 and from 3.652 to 7.227 mg/g in the upper and lower parts of* A. divaricatus*, respectively, and from 4.591 to 10.108 and from 3.834 to 9.079 mg/g in the upper and lower parts of* A. koreanum*, respectively (Table 4).

A previous paper reported that eleutherosides B (**1**) and E (**2**) contents were 0 and 1.804 mg/g in the stem of* A. divaricatus*, 0 and 1.016 mg/g in the root of* A. divaricatus*, 0.621 and 0.885 mg/g in the stem of* A. koreanum*, and 0.478 and 0.538 mg/g in the root of* A. koreanum*, respectively [11]. Our results showed a similar trend.

The best conditions to increase the eleutherosides B (**1**) and E (**2**) content in* A. divaricatus* were determined to be with 30 cm pinching, April 30 planting time, and a N-2P-K fertilizer ratio. In comparison, for* A. koreanum*, the eleutherosides B (**1**) and E (**2**) content was obtained with 30 cm pinching, March 30 planting time, and a 2N-2P-2 K fertilizer ratio. Moreover, the total eleutherosides B (**1**) and E (**2**) contents in the upper part of the plant were greater than in the lower part of* A. divaricatus* by pinching and fertilizer ratio and in the lower part of the plant they were greater than in the upper part of* A. divaricatus* by planting time. However, the result of the* A. koreanum* was the opposite. Therefore, the cultivation methods of* Acanthopanax* species should be established on an individual basis for each species considered. In conclusion, these results can be applied to optimize eleutherosides B (**1**) and E (**2**) production for harvesting from* A. divaricatus* and* A. koreanum* in nutraceutical, pharmaceutical, and cosmeceutical development.

## Figures and Tables

**Table 1:** The contents of eleutherosides B (**1**) and E (**2**) in *A. divaricatus *and* A. koreanum *divided by plant section.

Sample	Plant section∗	Content (mg/g)		
		**1**	**2**	Total
*A. divaricatus *	Ps-1	—	5.841 ± 0.054^a^	5.841 ± 0.054
	Ps-2	—	3.443 ± 0.147^c^	3.443 ± 0.147
	Ps-3	—	3.922 ± 0.019^b^	3.922 ± 0.019
	Ps-4	0.034 ± 0.013	3.078 ± 0.046^d^	3.112 ± 0.059
	Ps-5	—	2.466 ± 0.060^e^	2.466 ± 0.060

*A. koreanum *	Ps-1	3.229 ± 0.101^b^	3.722 ± 0.008^a^	6.951 ± 0.109
	Ps-2	2.482 ± 0.021^e^	1.857 ± 0.010^d^	4.339 ± 0.031
	Ps-3	2.601 ± 0.020^d^	1.822 ± 0.004^e^	4.423 ± 0.024
	Ps-4	3.080 ± 0.036^c^	2.365 ± 0.004^c^	5.445 ± 0.040
	Ps-5	3.801 ± 0.013^a^	3.559 ± 0.020^b^	7.360 ± 0.033

*Plant sections are as shown in Figure 1(a).

Data are presented as the mean ± SD (*n* = 3) in mg/g of the dried samples.

Means followed by the different superscript letters (a–e) are significantly different by Duncan's multiple range tests.

**Table 2:** The contents of eleutherosides B (**1**) and E (**2**) in *A. divaricatus *and* A. koreanum *cultivated by different pinching sites.

Sample	Pinching∗	Content (mg/g)		
		**1**	**2**	Total
*A. divaricatus *	Upper part			
	30 cm	Trace	6.827 ± 0.007	6.827 ± 0.007
	60 cm	Trace	6.301 ± 0.007	6.301 ± 0.007
	Lower part			
	30 cm	Trace	11.506 ± 0.012	11.506 ± 0.012
	60 cm	0.011 ± 0.008	7.847 ± 0.007	7.858 ± 0.015

*A. koreanum *	Upper part			
	30 cm	4.735 ± 0.521	3.112 ± 0.003	7.847 ± 0.524
	60 cm	2.188 ± 0.025	1.698 ± 0.008	3.886 ± 0.033
	Lower part			
	30 cm	3.099 ± 0.024	1.340 ± 0.003	4.439 ± 0.027
	60 cm	2.497 ± 0.008	1.664 ± 0.007	4.161 ± 0.015

*Upper and lower parts of the plant are as shown in Figure 1(b).

Data are presented as the mean ± SD (*n* = 3) in mg/g of the dried samples.

**Table 3:** The contents of eleutherosides B (**1**) and E (**2**) in *A. divaricatus *and* A. koreanum *planted at varying times.

Sample	Planting time	Content (mg/g)		
		**1**	**2**	Total
*A. divaricatus *	Upper part			
	March 30	Trace	6.491 ± 0.012	6.491 ± 0.012
	April 15	0.005 ± 0.002	4.285 ± 0.006	4.290 ± 0.008
	April 30	0.016 ± 0.001	6.510 ± 0.015	6.526 ± 0.016
	Lower part			
	March 30	—	3.769 ± 0.004	3.769 ± 0.004
	April 15	0.002 ± 0.001	3.985 ± 0.007	3.987 ± 0.008
	April 30	Trace	10.083 ± 0.012	10.083 ± 0.012

*A. koreanum *	Upper part			
	March 30	3.378 ± 0.012	2.315 ± 0.014	5.693 ± 0.026
	April 15	2.436 ± 0.015	2.110 ± 0.016	4.546 ± 0.031
	April 30	2.834 ± 0.007	2.349 ± 0.007	5.183 ± 0.014
	Lower part			
	March 30	3.590 ± 0.033	2.939 ± 0.013	6.529 ± 0.046
	April 15	1.887 ± 0.035	1.768 ± 0.012	3.655 ± 0.047
	April 30	3.225 ± 0.028	2.915 ± 0.024	6.140 ± 0.052

Plant divisions and data presentation are as shown in Table 2.

**Table 4:** The contents of eleutherosides B (**1**) and E (**2**) in *A. divaricatus *and* A. koreanum *cultivated with varying fertilizer ratio.

Sample	Fertilizer ratio	Content (mg/g)		
		**1**	**2**	Total
*A. divaricatus *	Upper part			
	N-P-K	0.006 ± 0.003	5.623 ± 0.001	5.629 ± 0.004
	2N-P-K	0.008 ± 0.001	5.280 ± 0.006	5.288 ± 0.007
	N-2P-K	Trace	6.905 ± 0.021	6.905 ± 0.021
	N-P-2K	0.018 ± 0.007	4.399 ± 0.022	4.417 ± 0.029
	2N-2P-2K	Trace	5.711 ± 0.007	5.711 ± 0.007
	Lower part			
	N-P-K	Trace	6.865 ± 0.007	6.865 ± 0.007
	2N-P-K	0.012 ± 0.006	3.640 ± 0.008	3.652 ± 0.014
	N-2P-K	0.013 ± 0.004	7.214 ± 0.022	7.227 ± 0.026
	N-P-2K	Trace	7.011 ± 0.008	7.011 ± 0.008
	2N-2P-2K	Trace	6.976 ± 0.006	6.976 ± 0.006

*A. koreanum *	Upper part			
	N-P-K	4.798 ± 0.030	3.101 ± 0.030	7.899 ± 0.060
	2N-P-K	3.203 ± 0.022	2.902 ± 0.030	6.105 ± 0.052
	N-2P-K	2.137 ± 0.024	2.454 ± 0.008	4.591 ± 0.032
	N-P-2K	3.473 ± 0.017	2.451 ± 0.012	5.924 ± 0.029
	2N-2P-2K	6.314 ± 0.007	3.794 ± 0.022	10.108 ± 0.029
	Lower part			
	N-P-K	2.083 ± 0.020	2.848 ± 0.027	4.931 ± 0.047
	2N-P-K	1.615 ± 0.039	2.219 ± 0.006	3.834 ± 0.045
	N-2P-K	2.707 ± 0.044	2.494 ± 0.005	5.201 ± 0.049
	N-P-2K	1.855 ± 0.018	2.088 ± 0.015	3.943 ± 0.033
	2N-2P-2K	4.918 ± 0.018	4.161 ± 0.008	9.079 ± 0.026

Plant divisions and data presentation are as shown in Table 2.
